# Miscellanies

**Published:** 1835-07-01

**Authors:** 


### MISCELLANIES.

### CASE OF DOUBTFUL TUMOUR ON THE THIGH. By Mr. ROBERT HOWARD, of Heptenstal, Yorkshire.

On April the 4th Mr. H. was desired to visit a young woman of the name of Helewell, of a strumous constitution.

On entering her room he was shown a tumour, about the size of a new born child's head; its dimensions appeared enormous; it occupied the upper portion of the thigh, its inferior margin seemed to terminate about where the lower edge of the sartorius muscle crosses the rectus femoris, laterally it extended from the vastus externus nearly to the gracilis, and superiorly it extended to the vicinity of Poupart's ligament. It was not a circumscribed tumour; but, on the contrary, was diffused, and unequal in its margin; in one place passing beyond the edge next to it, which gave it a peculiarly uneven appearance.

*History of the Case.*—Betty Helewell has been in the service of the Rev. Mr. Bentham, for the space of four years; and during the last 12 months of her service she was in a state of indisposition, the symptoms which manifested themselves, being debility, nausea, languor, paleness of the face, and occasionally pains about the sternum, accompanied by frequent chilliness, and constipation in the bowels.

Early after her arrival home, the catamenia became irregular, and the former symptoms became aggravated; another class of symptoms also made their appearance, viz. vertigo, palpitation of the heart and dyspepsia. She was also frequently troubled with a sense of weight at the inferior part of the abdomen. She has now been at home about two years. During the first eleven months, the catamenia were irregular; and during the subsequent twelve months, a complete suppression has existed.

Seven months ago, when the tumour was of small size, she felt pulsation in it, but not pain. It was less at one time than another, and occasionally it seemed almost to disappear. She several times noticed that, after a night's rest, it would become perceptibly diminished in size, but cannot say whether it would have receded or not by pressure.

From this period, it gradually enlarged, but she felt actually no pain, and it extended itself in an oblong direction, the pulsation in it became sensibly less, and for several months has not been perceptible.

On April the 4th I first examined the tumour, there was upon pressure a marked fluctuation, but pressure communicated no pain, she could bear almost any degree of pressure without complaining, and the tumour seemed in some degree to recede upon pressure, and the pressure removed, it obviously resumed its former magnitude.

*Peculiarities of the Tumour at its greatest magnitude.*—It has been previously observed that the margins of the tumour were irregular; it was moreover oblong, and in some degree seemed to follow the track of the femoral artery; the apex was not conical or pointed, but broad; about the centre of the top of the tumour there was slight discolouration, and a slough was forming. It receded, in some degree, on pressure. Slight pain was felt in the swelling, but, its temperature was not increased beyond that of the corresponding thigh. She could with facility have performed any duty in the house, had not the magnitude of the tumour been an impediment, no symptomatic fever manifested itself, and her nocturnal repose was not disturbed by the tumour.

She felt at this period no pulsation in the tumour. With symptoms so equivocal, with a tumour situated upon the course of the femoral artery; and taking into consideration the other concomitant circumstances, would it have been prudent to have made an opening into the tumour?

1st. Is it an abscess arising from a scrophulous affection of the lymphatics of the thigh?

2nd. Is it a diffused aneurism of the femoral artery?

3rd. Is it a critical abscess?

We need not detail Mr. Howard's speculations on these points, for the following reason.

On the 18th of this month the tumour burst, and I believe not less than five gills of a fluid were discharged. It was serous, and of a moderate red colour; indeed it much resembled the fluid parted with during menstruation; the smell was not particularly offensive, and some two or three times I had portions of matter to remove from the orifice by the forceps; they were of a cheese colour, and about the consistence of the coagulable lymph of the blood.

Up to the present time (April the 28th) not one unfavourable symptom has developed itself, and to all appearance the orifice will soon be healed. I may here add, that a degree of pressure is kept upon the part by means of a linen roller, passed around the body and down the thigh.

P.S.—*April 20th.* Her pulse are almost natural, her appetite is good, and her rest is refreshing; the symptoms enumerated in the early part of this paper have disappeared, and her complexion is much improved, whilst at the same time, her countenance indicates the return of health.

---

### ACALEPHÆ.

In the first number of the new Cyclopædia of Anatomy and Physiology (a work which we think, commences with very fair prospects of success) there is an interesting account of a very curious class of animated beings, holding a very low grade in the extensive scale of creation, yet exciting the wonder of the anatomist and the naturalist, much more than higher links in the chain of God's works. This is the ACALEPHÆ, or sea-jelly, sea-nettle, Portuguese man of war, &c. known, from the property which many of the animals possess of stinging those parts of our body which come in contact with them. We can only make room for a short extract, to shew the manner in which Dr. Coldstream has handled his subject in this article.

" On many accounts the acalephæ are objects of extreme interest to the anatomist and physiologist. They have occupied the attention of the most learned naturalists of every age, from the time of Pliny until the present day; their numbers are, perhaps, greater than those of any other class of marine animals: they exist in all seas; and yet we remain very ignorant with regard to several points in their structure and history. The peculiar nature of their tissues, the singular arrangements of their organs, the anomalies in their functions, present as many objects of interesting inquiry to the physiologist, as the wonderful variety and striking elegance of their forms, and their splendid colouring present to the admiration of the naturalist. Peron, in his animated description of the *Medusæ*, observes, ' among the animals of this family we find the most important functions of life performed in bodies which offer to the eye little more than a mass of jelly. They grow frequently to a large size, so as to measure several feet in diameter; and yet we cannot always determine what are their organs of nutrition. They move with rapidity, and continue their motions for a long time; and yet we cannot always satisfactorily demonstrate their muscular system. Their secretions are frequently very abundant, and yet the secreting organs remain to be discovered. They seem to be too weak to seize any vigorous animal, and yet fishes are sometimes their prey. Their delicate stomachs appear to be wholly incapable of acting upon such food, and yet it is digested within a very short time. Most of them shine at night with great brilliancy, and yet we know little or nothing of the nature of the agent which produces so remarkable an effect, or of the organs by which it is elaborated. And, lastly, many of them sting the hand which touches them; but how, or by what means, they do so still remains a mystery.' It is, therefore, but a very imperfect account of the anatomy and physiology of this class that can be at present given."

The anatomy of the various species is illustrated by numerous wood-cuts, and the researches of Dr. Coldstream are very creditable to himself and the work to which he contributes.

---

### ON THE NUMBER OF THE PULSATIONS OF THE HEART IN THE FŒTUS BY G. O. FLEMING. M.D. F.L.S. Physician Accoucheur to the Pancras Infirmary.

I have reason to think that the account given of the number of pulsations of the heart in the fœtus, by Laennec and M. Kergaradec is erroneous. These observers speak of a number of *double pulsations,* double the number of the pulse of the mother. I listened to the fœtal heart with F. Cumin, during my late residence in Glasgow, and found the number of pulsations to be 140. But these were not *double* but *single* pulsations; and, from a subsequent observation, I am now persuaded that each two pulsations were, in fact, one *double pulsation;* and on a recent occasion, I had an opportunity of counting the pulse in an infant just born, but which had not breathed; it was 70. The moment gasping and breathing took place, the pulse became much quicker; it was irregular at first, but soon became 140 in a minute.

I think we may conclude from these facts, that the fœtal pulse is doubled immediately upon the perfect establishment of respiration.

*May* 30, 1835.

---

### Medical Associations.

We perceive that a plan is in process of organization, in several counties, and will, doubtless, be followed out in all, of forming associations of all descriptions of medical practitioners, for the avowed purpose of maintaining the respectability of the profession, and preventing or discouraging degradation of it by members who are more solicitous of acquiring a wretched subsistence than of adding or supporting the dignity of a liberal profession. At all times there were some medical men who evinced no very nice sense of distinction between trade and science. But now, the distinction is likely to be entirely obliterated by the competition which is excited, or being called forth by the system of "farming the poor" in the doctoring way, in the same style as contracts for biscuit and salt pork used to be advertized during the war—and precious supplies of these articles were the consequence!! Many a legion of poor seamen went to an untimely and watery grave by the villainous contracts for cheap provisions; yet still there was something like reason and justice in advertising for articles, in order that the lowest bidder might have the contract. But when this principle is applied to medical skill and attention, as well as to the very drugs which are employed, it "bangs bannagher," as the facetious O'Connell says, and were not the subject of a most serious, not to say melancholy nature, we would be inclined to laugh at the proposition, as the offspring of some lunatic imagination. It is, however, but too true, and will continue, unless commissioners and church-wardens are shamed out of the system by the general expression of indignation among all the respectable orders of medical society. We cannot, therefore, permit this number of the Journal to go forth, without expressing our approbation of the associations which are now forming, and of encouraging the formation of others, where plans are not yet in progress of organization. The following resolutions adopted by the medical association in Kent, may serve as a model, or at least an example, for the resolutions of other countries.

### Resolutions.

"1. That the system of parishes contracting for attendance upon their sick poor, is in principle highly objectionable, and practically injurious, both to medical men and their pauper patients, *unless a fair remuneration be given, on the principle of Friendly Societies, as framed by the Rev. J. T. Beecher, of Southwell.*

2. That the continuance of that system, under the direction of the new poor law commissioners, is viewed with deep regret by this meeting.

3. That the terms for medical attendance upon, and furnishing medicines to the sick poor, proposed by the guardians of several unions recently formed in the eastern division of this county, are by no means proportionate to the duties required of medical officers by the commissioners, and to the expense, and moral and legal responsibility, they must necessarily incur.

4. That it appears to this meeting that the other salaried officers, under the new poor law act, from commissioners downwards, are far more liberally paid, in proportion to the duties assigned them and their rank, than it is proposed to pay the parochial medical officers.

5. That in adopting the foregoing resolutions, the members of the medical profession here present are actuated not merely by the desire of maintaining the just interests and the dignity of the profession, but also of consulting the interest of the rate-payer, as well as the benefit of their poorer neighbours.

Lastly, That an association be formed, to be called the East Kent Medical Association. That the principal objects of this association be to preserve the privileges and support the credit of surgeons and apothecaries, to promote fair and liberal practice, and prevent abuses in the profession; and to adopt such measures as may appear best calculated to effect those ends."

---

### Medical Reform.

We have not amused ourselves or our readers, of late, with any of the numerous rumours, intelligence, &c. &c. so liberally scattered among the public by the press, respecting *internal* reforms in certain great bodies. It is now pretty evident that they were all "baseless fabrics of a vision." We never expect a liberal reformation spontaneously adopted in any one of our medical establishments; and therefore we are not at all sorry that the attempts to blind us with abortive plans, that would confer no real benefit on the profession at large, have vanished into air—thin air. The great events that are now engaging the attention of legislators will, in all probability prevent any parliamentary measures being entertained on the subject of medical reform during the present year. The *stationaries* may construe this procrastination (and we have no doubt that they will do so) into a final abandonment of all legislative interference with the present order of things in the republic of medicine. In the minds of most others, however, it will appear tolerably probable that the sweeping reform which is about to take place in municipal corporations, will not leave the medical corporations very long as anomalies in the municipal code. But should the legislature defer, for some years, the expected amelioration of our medical laws and institutions, the rapid growth of public opinion will render the final result not the less certain because it is delayed. We have little doubt, in the mean time, that Mr. Warburton is *digesting* his plan of reform, and that, in due and good time, it will be brought under the consideration of Parliament. All pretended codes and regulations so confidently prognosticated by several of our contemporaries, are, "*voces et preterea nihil.*"

---

